# Knowledge, Attitudes and Practices Regarding Rift Valley Fever Among Livestock Traders in the Alaotra Mangoro Region, Madagascar

**DOI:** 10.3390/tropicalmed11050136

**Published:** 2026-05-16

**Authors:** Félix Alain, Botovola Miraimila, Véronique Chevalier, Peter N. Thompson

**Affiliations:** 1 Department of Veterinary Tropical Diseases, Faculty of Veterinary Science, University of Pretoria, Pretoria 0002, South Africa; 2 National Institute of Public and Community Health (INSPC), Antananarivo 101, Madagascar; miraymila90@gmail.com; 3 Faculty of Medicine, University of Antananarivo, Antananarivo 101, Madagascar; 4 Centre for International Cooperation in Agricultural Research for Development (CIRAD), Antananarivo 101, Madagascar; veronique.chevalier@cirad.fr; 5 Department of Production Animal Studies, Faculty of Veterinary Science, University of Pretoria, Pretoria 0002, South Africa; peter.thompson@up.ac.za

**Keywords:** knowledge attitudes and practices, livestock traders, Madagascar, one health, rift valley fever

## Abstract

Rift Valley fever (RVF) is a viral zoonosis endemic in Madagascar, threatening human and animal health as well as the economy. Trade-related livestock movements are a major factor in the spread of RVF virus. While previous RVF research in Madagascar has focused on farmers or general ecology, this study is the first to specifically target livestock traders, the primary drivers for long-distance viral spread, in the Alaotra Mangoro endemic hotspot. This study aimed to assess the level of knowledge, prevailing attitudes and current practices regarding RVF among people engaged in livestock trade in the Alaotra Mangoro region, as well as the factors associated with these KAPs. A descriptive and analytical cross-sectional survey was conducted among 406 livestock traders in five districts of the Alaotra Mangoro region, using a structured questionnaire. A multi-stage sampling approach was employed, utilising purposive selection of markets followed by snowball sampling to reach informal traders often missed by traditional surveys. Generalised linear mixed models were used to analyse factors associated with KAPs regarding RVF. Awareness of RVF was very low (only 18.5% respondents had heard of it), with significant regional disparities (0% in Anosibe An’Ala versus 51.6% in Moramanga). Veterinarians (15.5%), family (12.8%), radio (9.6%) and neighbours (9.6%) were the main sources of information. Understanding of symptoms and modes of transmission (particularly mosquito bites) was limited. Higher levels of education (OR = 181.6; 95% CI: 29.9–1123.7; *p* < 0.001) and older age (50–60 years) were associated with better knowledge. Proactive attitudes were scarce (21.4%), although more than half (53.4%) believed that RVF is a real disease. Perception of personal risk and the contribution of livestock trade to the spread of the disease was low. However, confidence in animal vaccination was relatively high (60.3%). Preventive practices were highly inadequate. The majority did not wear protective equipment when handling sick animals (94.6%) and rarely avoided touching aborted foetuses (12.6%). Less than half (48.3%) expressed a willingness to report sick or dead animals, and nearly half admitted to having sold or purchased sick livestock (49.5%). Cooking meat (95.1%) and using mosquito nets (74.1%) were the only well-established practices. More than half of respondents (57.9%) lived more than 5 km from veterinary services, and cost was the most frequently cited barrier to consultation. Participation in awareness campaigns was virtually non-existent (5.4%). Results revealed critical gaps in KAP that may contribute to the persistence of RVF. A “One Health” approach is imperative, integrating human, animal and environmental health.

## 1. Introduction

Rift Valley fever (RVF) is a mosquito-borne viral zoonosis that represents a persistent threat to public health and the socio-economic stability of Madagascar [1]. The disease is caused by the RVF virus (RVFV), a single-stranded RNA pathogen belonging to the genus *Phlebovirus*, family *Phenuiviridae*, order *Hareavirales*, and class *Bunyaviricetes* [2,3,4]. In ruminants, infection may result in high neonatal mortality and “abortion storms,” while human clinical presentation ranges from mild to severe, sometimes with fatal outcomes [1,2,5]. Effective disease control is frequently hindered because diagnosis based solely on observable signs in livestock is insufficient, frequently leading to misdiagnosis and underreporting [5].

First identified in Madagascar in 1979, RVF has since become endemic, with major outbreaks recorded in 1990–1991, 2007–2008 and 2021 [6,7]. Serological evidence of persistence of RVFV and continued seroconversion was reported between 2008 and 2021 [5,7,8]. The Alaotra Mangoro region was one of the most affected regions in both the 2008 and 2021 outbreaks, suggesting persistent circulation of the virus during the interepidemic period [5,9]. Continued viral activity is driven by a complex interaction between the island’s unique ecological conditions, an abundance of competent mosquito vectors, and human behaviour [10]. Specifically, trade-related livestock movements are a well-recognised mechanism for the introduction and long-distance spread of RVFV in Madagascar [11].

Despite collaborative efforts from the WHO, WOAH, and FAO alongside Malagasy authorities, RVF remains a major concern on the island [3,12]. Local control strategies often rely on Proximity Agents in Animal Production and Health (APPSA) to extend community-level veterinary coverage [13]. Optimal prevention requires an understanding of the Knowledge, Attitudes, and Practices (KAP) of key stakeholders, such as livestock traders. However, research explicitly connecting RVF with traders in endemic hotspots like Alaotra Mangoro remains scarce. This lack of reliable information limits the efficacy of public health interventions and the development of effective epidemiological approaches.

This study is grounded in a conceptual framework where improved knowledge of RVF epidemiology is expected to shape proactive attitudes and risk perceptions, which in turn drive the adoption of safer animal handling, reliable disease reporting, and responsible trade behaviours essential for effective surveillance and outbreak control. This approach aligns with One Health initiatives. It recognises that consideration of the interaction between human, animal, and environmental health is vital for economic resilience [3].

## 2. Materials and Methods

### 2.1. Study Framework and Location

**Figure 1:**
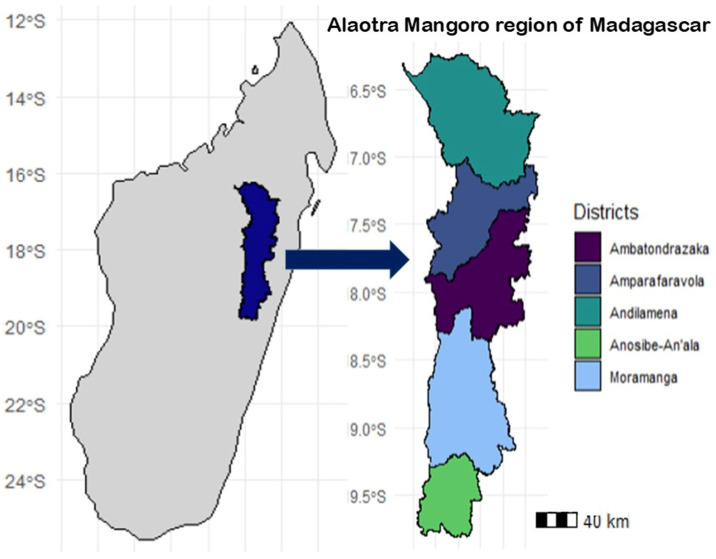
Geographic location of the five study districts within the Alaotra Mangoro region, Madagascar.

This study was conducted in the Alaotra Mangoro region (Figure 1), located in the central-east of Madagascar. The Alaotra Mangoro region was prioritised as it is among the top four regions most affected by RVF, having experienced heightened public awareness following the 2021 RVF outbreak [14,15]. The region, home to approximately 1.256 million people in 2018 [15], is known for its diverse agricultural production and unique ecosystem. All five districts of the region were included in the survey, namely Amparafaravola, Andilamena, Ambatondrazaka, Moramanga, and Anosibe An’Ala. In these study districts, as in the rest of the country, no vaccination against RVF is practised.

### 2.2. Study Design and Period

A descriptive and analytical cross-sectional survey was carried out to document KAP regarding RVF among individuals involved in buying and selling livestock. Data collection for the study was carried out between 16 July and 26 July 2025.

### 2.3. Study Population and Ethical Considerations

The study population consisted of individuals whose primary economic activity involves the commercial exchange of livestock, categorised by their secondary roles (e.g., butcher-traders or breeder-traders) to allow nuanced analysis. Participants had to reside in the Alaotra Mangoro region, and be 18 years or older. All individuals were required to provide informed, oral consent before enrolling into the study.

### 2.4. Sampling Methods and Sample Size

A multi-stage, mixed-method sampling strategy was adopted to ensure diversity and representativeness. All five districts within the Alaotra Mangoro region were included to capture regional differences in RVF exposure, perceptions, and practices. Traders were recruited during peak hours at major livestock markets. In the absence of a formal registry, market leaders facilitated initial contacts, followed by snowball sampling to identify potential respondents.

A key component of this approach was the use of snowball sampling [16,17]. This technique was critical for accessing traders operating outside formal markets or those in hard-to-reach or informal settings. It therefore ensured the participation of individuals from diverse trading environments. Snowball sampling was initiated after initial contact was made through markets or local officials, and confidentiality and informed consent were maintained throughout this process.

The minimum sample size (*n*) required to estimate the proportion (*p*) of livestock traders employing specific biosecurity measures was calculated using a desired confidence level of 95% (Z = 1.96), an estimated proportion of *p* = 0.5 (chosen to maximise the sample size, as the true proportion was unknown), and a margin of error of L = 0.05. The calculated minimum sample size was 385 livestock traders.

### 2.5. Variables and Scoring

The study focused on four main categories of variables: demographic characteristics, knowledge of RVF, attitudes toward RVF, and practices related to RVF prevention. Independent variables included demographic features (e.g., age, gender, education level, and place of residence). Dependent variables encompassed the three KAP domains.

To allow for quantitative analysis, a binary scoring system was applied to the attitude (7 items) and practice (9 items) responses. Each positive response was allocated a score of 1. Spontaneous awareness was assessed by whether the participant had heard of RVF (Yes = 1, No = 0). A composite knowledge score (0–20) was then derived from 20 specific items: clinical signs in animals (6 items), symptoms in humans (7 items), transmission routes (5 items), and seasonality (2 items). Each correct unprompted mention was allocated 1 point. For multivariable analysis, ‘adequate knowledge’ was defined as a score of 1 or higher, distinguishing those who could identify at least one epidemiological feature from those with no knowledge [18,19,20].

The classification thresholds for the KAP domains in the study were established such that a “good attitude” was characterised by a score of 6 or higher out of 7, whereas a poor attitude was defined by any score lower than 6. Regarding the classification of practices, “good practice” was defined as achieving a score of 8 or more out of 9, while a poor practice designation was assigned to any score below 8.

### 2.6. Data Collection and Management

Data collection was executed through in-person surveys using a structured questionnaire. The questionnaire was pre-tested among 20 traders in a neighbouring region to ensure clarity. Interviews were conducted face-to-face in the local Malagasy dialect using tablets equipped with the KoBoCollect programme to minimise comprehension bias and ensure data reliability [21]. This electronic data capture facilitated enhanced data security, real-time error identification, and reliable capture. Supervisors monitored the interviewers to ensure adherence to due processes and maintain data reliability and completeness. Following collection, the dataset was downloaded, cleaned, and imported into R Studio (version 4.4.3) for processing and analysis.

### 2.7. Data Analysis

Frequencies and percentages for categorical variables, and means and standard deviations for continuous variables, were computed to provide an initial overview of respondent characteristics and basic RVF KAP indicators.

Bivariate analysis was performed using Chi-square or Fisher’s exact tests for categorical variables, and Student’s *t*-tests for continuous variables, to examine initial associations with the binary KAP outcomes. Odds ratios (ORs) with 95% confidence intervals (CIs) were calculated as measures of association. Variables found to be associated with the outcome at a significance level of *p* < 0.20 were considered, along with theoretical relevance, for subsequent multivariable modelling.

The multivariable analysis employed Generalised Linear Mixed-Effects Models (GLMMs) to account for the hierarchical structure of the data and the clustering of observations within districts. Collinearity between explanatory variables was assessed using the Variance Inflation Factor (VIF); if VIF >5, one variable was removed to ensure model stability.

Three separate GLMMs [22] were fitted using the “*glmer()*” function of the “*lme4*” package in R: one for knowledge, one for attitudes, and one for practices. Each model included fixed explanatory variables, such as gender, education level, household size, age, distance to veterinary services, etc. Random effects were incorporated at the district level to control for unobserved heterogeneity. The structure using district as a random effect was retained as it provided a better statistical fit (lower AIC value) compared to an equivalent fixed effect model. Statistical models reported adjusted odds ratios (aORs) with 95% CIs. A two-tailed *p*-value of <0.05 was considered statistically significant.

### 2.8. Ethical Considerations

Ethical approval was granted by the Ethical Evaluation Committee for Biomedical Research (Comité d’Évaluation Éthique pour la Recherche biomédicale—CEER) of the National Institute of Public and Community Health (Institut National de Santé Publique et Communautaire—INSPC) (Ref: 12/2025-CEERINSPC/IRB). Approval was also obtained from the Research Ethics Committees of the University of Pretoria, Faculty of Veterinary Science (Ref: REC165-24) and Faculty of Humanities (Ref: HUM005/0225). Participants provided verbal informed consent following a detailed explanation of the study objectives. Anonymity was strictly maintained, with all identifying details removed from the final dataset.

## 3. Results

### 3.1. Study Population

A total of 406 questionnaires were collected from livestock traders across the five districts of the Alaotra Mangoro region. The distribution of participants was as follows: Moramanga (22.9%, n = 93), Andilamena (22.4%, n = 91), Ambatondrazaka (21.2%, n = 86), Anosibe An’Ala (17.2%, n = 70), and Amparafaravola (16.3%, n = 66). Data collectors administered the questionnaire directly through in-person interviews following a process of voluntary, verbal informed consent. A total of 410 traders were approached, and 406 provided full informed consent and completed the interview, representing a 99.0% participation rate.

### 3.2. Socio-Demographic Characteristics

The respondents had a mean age of 35.6 years (range: 18 to 67 years), with the largest age group being individuals between 18 and 29 years old (33.7%). The livestock trading sector was predominantly male (93.1%), a trend observed across all five surveyed districts.

Regarding social roles (see Table 1), 81.5% of respondents were married, and 80.5% identified as the head of their household. Educational attainment was generally low: 18.2% of traders reported having no formal schooling, while 47.0% had completed only primary education. Only 6.9% of participants had reached a university or college level of education.

### 3.3. Economic Profile and Livestock Engagement

Livestock was the primary income source for 81.3% of respondents (see Table 1), and 94.8% derived income from trade. The majority of participants (65.0%) identified “trader” as their main occupation. Financial incentives were cited by 45.1% of respondents as the main motivation for entering the livestock business.

Regarding the ownership of communication and information tools among the surveyed livestock traders, radios were owned by 80.5% of participants, while 61.1% owned a cell phone and 24.6% owned a television. Apart from trading, respondents were actively involved in rearing of various species, primarily cattle (91.9%), followed by pigs (36.0%) and sheep (9.6%).

### 3.4. Knowledge of RVF Among Livestock Traders

**Figure 2:**
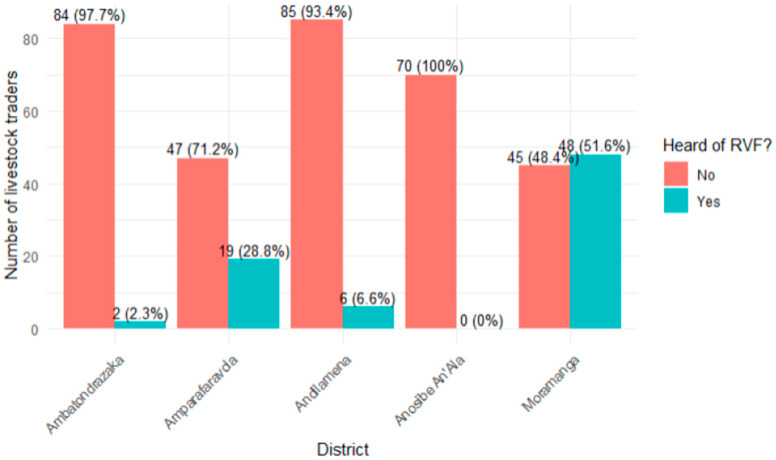
Awareness of RVF among livestock traders, by district, Alaotra Mangoro, Madagascar (N = 406).

Of the 406 livestock traders surveyed, 18.5% (75/406) had heard of RVF (Figure 2), while 81.5% had no prior awareness of the disease. Awareness levels varied significantly by district (*p* < 0.001); the highest level was recorded in Moramanga (51.6%), followed by Amparafaravola (28.8%), Andilamena (6.6%), and Ambatondrazaka (2.3%). In Anosibe An’Ala, none of the respondents (0%) reported having heard of the disease.

Among the 75 participants who had heard about RVF before (Table 2), 63 (15.5%) mentioned hearing about it from veterinarians, 52 (12.8%) from family members, 39 (9.6%) from the radio, and 39 (9.6%) from neighbours. Specific knowledge regarding clinical signs and transmission was limited. Regarding signs in animals, 15% identified sudden abortions as a sign of RVF, while other reported signs included deaths (13.5%) and nasal discharge (9.4%). Regarding symptoms in humans, the most frequently cited were haemorrhage (9.4%) and death (7.1%). Regarding transmission to humans, 11.6% cited eating raw or undercooked meat, 8.9% mentioned contact with an aborted foetus, and only 1.2% of respondents identified mosquito bites as a mode of transmission.

In terms of seasonality, 8.9% of the 75 traders who had previously been aware of RVF identified the rainy season as the period of highest frequency for RVF. However, only 3.9% of respondents reported adapting their practices during high-risk periods.

### 3.5. Attitude of Livestock Traders Towards RVF

**Figure 3:**
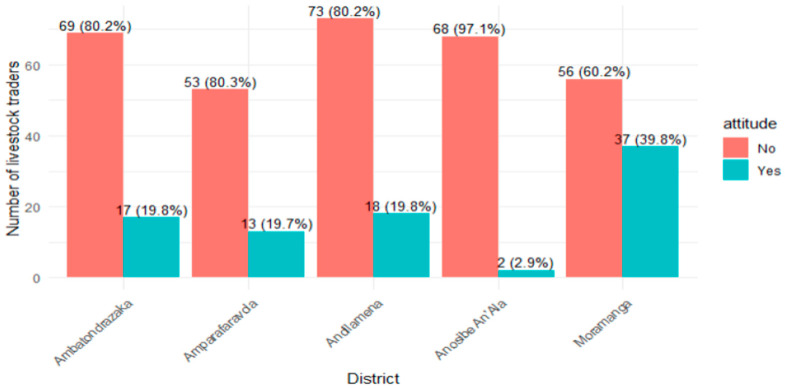
Proportion of livestock traders demonstrating positive attitudes toward Rift Valley fever prevention by district (N = 406).

The general level of awareness and concern regarding RVF among livestock traders in the Alaotra Mangoro region was low (Figure 3), with only 21.4% of respondents classified as having a “good attitude” (score ≥ 6 out of 7). Significant disparities were observed between districts (*p* < 0.001); Moramanga recorded the highest proportion of positive attitudes (39.8%), while Anosibe An’Ala recorded the lowest (2.9%).

Regarding perceptions of the disease, 53.4% of participants agreed that RVF is a real disease, while 46.6% disagreed. Among those acknowledging the threat, 24.6% considered it “very dangerous,” 31% viewed it as a moderate danger, and 18.7% stated it was not dangerous at all. The belief in zoonotic transmission (animal-to-human) was held by 48.3% of respondents, whereas 30% were unsure and 21.7% responded negatively.

Perceptions of personal risk (Table 3) were divided: 34.5% of traders felt at risk, 33.7% did not, and 31.8% were uncertain. The perceived risk was highest in Moramanga (51.6%) and lowest in Amparafaravola (24.2%).

Livestock vaccination practices were common, with 88.9% (361/406) of traders reporting having vaccinated their animals against livestock diseases (although this did not include RVF). General confidence in animal vaccination was expressed by 60.3% of participants, while 10.6% were not confident and 29.1% remained unsure. When asked whether livestock trade contributes to the spread of RVF, 24.6% agreed, 32.8% disagreed, and 42.6% were uncertain.

### 3.6. Practices Regarding RVF Among Livestock Traders

**Figure 4:**
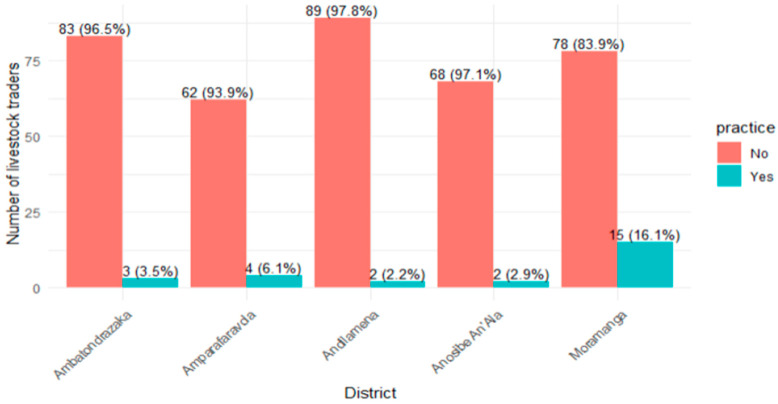
Percentage of participants with good prevention practices related to RVF, by district, Alaotra Mangoro, Madagascar (N = 406).

The overall level of preventive practices (Figure 4) related to RVF among livestock traders in the Alaotra Mangoro region was low, with only 6.4% of respondents reporting “good practice” (score ≥ 8 out of 9). Significant regional variations were observed (*p* < 0.001), with the highest proportion of good practices recorded in Moramanga (16.1%), while other districts ranged from 2.2% to 6.1%, the lowest being in Andiamena.

Specific preventive behaviours and high-risk practices (Table 4) included:

Personal protection: a large majority of respondents (94.6%) reported not using any form of protection when handling sick animals. The use of gloves was the most cited measure among the 5.4% who utilised protection.

Vector control: 74.1% of participants reported sleeping under a mosquito net. The primary reasons cited for non-use (25.9%) were heat/discomfort (14.5%) and habit or personal preference (13.3%).

Handling of infected materials: a majority (87.4%) of traders did not avoid touching aborted foetuses, and 92.1% of respondents reported taking no proactive steps to avoid contact with animal blood or fluids.

Reporting and surveillance: less than half of the participants (48.3%) indicated a willingness to notify authorities regarding sick or dead animals. Willingness was highest in Andilamena (63.7%) and lowest in Anosibe An’Ala (10%).

Trade and slaughtering: 49.5% of respondents acknowledged having purchased or sold sick cattle. Additionally, 42.9% admitted to sometimes slaughtering sick animals, primarily for sale or personal consumption (42.6%).

Consumption habits: while 95.1% of traders reported cooking meat properly to prevent disease, 14.8% admitted to consuming unboiled (raw) milk, and 42.4% had eaten meat from sick animals.

### 3.7. Role of Government and NGOs in RVF Awareness

Trust in sources of information regarding RVF (Table 5) varied significantly across districts (*p* < 0.001). Private veterinary services were identified as the most trusted source, with 76.8% of respondents expressing confidence in them, particularly in Moramanga (96.8%) and Ambatondrazaka (84.9%). In contrast, 38.2% of traders trusted information from the government, and 13.3% trusted NGOs. In Anosibe An’Ala, 35.7% of respondents were uncertain about which source to trust.

Participation in RVF training or awareness campaigns was 5.4% (22/406) overall. The highest participation rates were in Moramanga (15.1%) and Amparafaravola (10.6%), while participation was nearly non-existent in other districts. Among participants, 5.2% identified veterinary services as the organisers, while only 0.2% cited the government and none cited NGOs.

### 3.8. Access to Veterinary Services and APPSA Service

Geographic access to veterinary care (Table 6) showed significant regional disparities (*p* < 0.001). Overall, 57.9% (235/406) of respondents reported that the nearest veterinary or APPSA facility was more than 5 km away. This proportion was greatest in Anosibe An’Ala (84.3%) and Moramanga (68.8%).

Regarding clinical consultations, 53.4% of traders reported “sometimes” consulting a veterinarian when animals are sick, while 27.3% “always” did so. In Anosibe An’Ala, 41.4% reported “never” seeking veterinary services. The primary barrier to consultation was cost or lack of financial means (9.1%), followed by distance (2.5%) and lack of trust (2.0%). Among those who “always” consulted, 103/111 (93%) utilised veterinarians only, while 7% used both APPSA and veterinarians.

### 3.9. Reported Economic Losses and Market Uncertainty

Of all respondents, 18.2% (74/406) reported experiencing economic losses due to uncertainty in the past five years. The distribution (see Table 7) was uneven, with the highest prevalence in Anosibe An’Ala (24.3%), Amparafaravola (22.7%), and Moramanga (22.6%), compared to 3.3% in Andilamena (*p* = 0.001).

The most frequent type of loss was animal death (16%), which was most prevalent in Anosibe An’Ala (24.3%). Other reported impacts included market bans (5.4%) and a decline in income due to reduced productivity or trade (5.2%).

### 3.10. Factors Associated with Adequate Levels of Knowledge, Attitude and Practices Towards RVF

**Figure 5:**
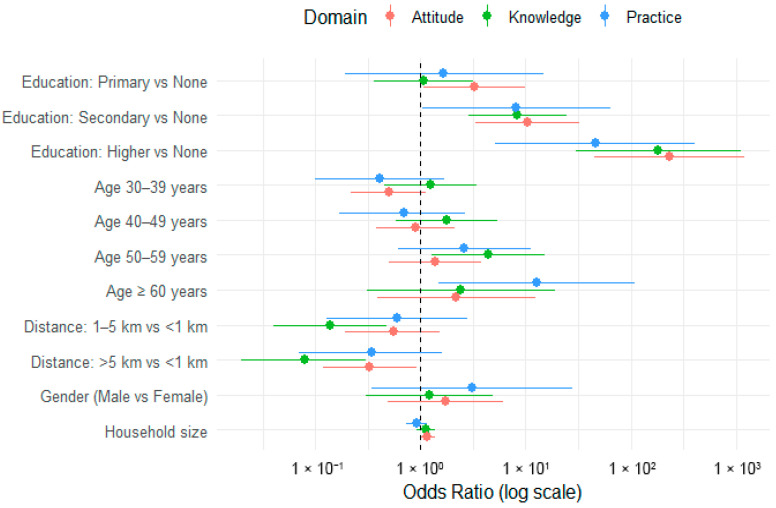
Forest plot of adjusted odds ratios for socio-demographic factors associated with Knowledge, Attitude, and Practice (KAP) regarding Rift Valley fever.

A total of 12 candidate explanatory variables were initially screened in a preliminary bivariate analysis: age, gender, marital status, role in the household, household size, educational level, main source of income, main occupation, motivation for livestock trading, years in business, frequency of trade, and distance to veterinary services. Five predictor variables—gender, education level, household size, age, and distance to veterinary services—were selected (see Figure 5) for the final multivariable models based on statistical significance (*p* < 0.05) and theoretical relevance. Three separate GLMMs (see Table 8) were developed for Knowledge, Attitudes, and Practices, incorporating district as a random effect.

#### 3.10.1. Factors Associated with Knowledge

Education level emerged as the primary factor associated with RVF knowledge. Traders with a secondary education were 8.4 times more likely to be aware of RVF compared to those with no formal education (95% CI: 2.8–24.8; *p* < 0.001), while those with higher education had even greater odds (aOR = 181.6; 95% CI: 29.9–1123.7; *p* < 0.001).

Distance to veterinary services was a strong negative predictor. Individuals living 1–5 km from a veterinarian were less likely to be aware of RVF than those living <1 km away (aOR = 0.1; *p* = 0.002), with a further decrease in odds for those living >5 km away (OR = 0.1; *p* < 0.001). Regarding age, only the 50–59 age group showed a positive association with knowledge compared to those <30 years (aOR = 4.4; *p* = 0.019).

#### 3.10.2. Factors Associated with Attitudes

Higher education levels were significantly associated with positive attitudes toward RVF prevention. Compared to traders with no schooling, the odds of having a positive attitude increased with primary education (aOR = 3.3; *p* = 0.039), secondary education (aOR = 10.4; *p* < 0.001), and higher education (aOR = 233.7; *p* < 0.001).

Traders living >5 km from the nearest veterinary service were less likely to hold positive attitudes compared to those within 1 km (aOR = 0.3; 95% CI: 0.1–0.9; *p* = 0.036).

#### 3.10.3. Factors Associated with Practices

Secondary and higher education were linked to positive preventive practices. Traders with a secondary education had 8.2 times higher odds (95% CI: 1.04–64.1; *p* = 0.045) and those with higher education had 45.8 times higher odds (*p* < 0.001) of implementing prevention measures compared to those with no education.

Age was also a significant factor, as traders aged ≥60 years demonstrated substantially increased odds of positive/good attitude (aOR = 12.6; 95% CI: 1.5–108.3; *p* = 0.021) compared to those aged 18–29 years. Gender, household size, and distance to veterinary services showed no significant association with practice levels.

## 4. Discussion

This study aimed to assess the level of knowledge, attitude and practices of livestock traders regarding RVF in the Alaotra Mangoro region of Madagascar. In Madagascar, livestock trade is recognised as a primary factor in the spread of RVFV, where animal movements between regions significantly increase the risk of new outbreaks [11]. The current results indicate a concerning situation characterised by low levels of awareness, negative or indifferent attitudes, and high-risk behaviours that likely facilitate the persistent circulation of this zoonotic pathogen [23]. Our finding of 18.5% awareness is significantly lower than the 90% observed in Uganda and 97.6% in Tanzania, suggesting a critical need for basic sensitization of livestock traders in Madagascar to RVF [19,24,25,26,27,28,29].

### 4.1. Knowledge of RVF Among Livestock Traders

Overall awareness of RVF was strikingly low, with only 18.5% of respondents having previously heard of the disease. A significant regional disparity was observed: awareness reached 51.6% in Moramanga but was completely absent (0%) in Anosibe An’Ala. The higher awareness in Moramanga may be associated with its status as a central trading hub; however, further investigation is required to determine if this is due to historical experience or recent interventions [10,30]. This centrality has resulted in greater targeted veterinary focus, leading to better sustained knowledge transfer compared to more isolated districts [9]. While awareness is typically higher in other nations, such as Tanzania (97.6%) and Uganda (90%), being aware of the disease does not necessarily equate to a good understanding of it [24,27].

The poor recognition of clinical signs remains a major barrier to early detection. Only 15% of traders identified sudden abortions, a crucial warning sign, as a sign of RVF in animals [5]. Knowledge regarding transmission was even more restricted; only 1.2% of respondents mentioned mosquito bites, reflecting a widespread ignorance about the role of vectors [29]. Furthermore, the poor recognition of severe human symptoms, particularly haemorrhage, may significantly delay essential healthcare seeking [27].

### 4.2. Attitudes and Perceptions of Risk

Traders’ attitudes were often contradictory and underestimated the potential threat of the disease. Only 53.4% of participants believed RVF was a real disease, a rate much lower than that observed in Tanzania (90.3%) or Sudan (80%) [24,26]. Furthermore, only 24.6% of respondents considered RVF to be “very dangerous,” suggesting a limited perception of risk [28]. This low perceived danger is a major obstacle to the adoption of biosecurity measures in the livestock trade [19]. However, the high level of trust in private veterinary services (76.8%) and confidence in animal vaccination (60.3%) provides a positive basis for strengthening future prevention campaigns [9,13].

### 4.3. Risky Practices and Economic Drivers

Preventive practices were highly inadequate, with only 6.4% of participants reporting appropriate behaviours. Risky behaviours were prevalent: 94.6% of traders did not use protective equipment when handling sick animals, and 49.5% admitted to buying or selling sick livestock. In Madagascar, Zebu cattle are essential for draft power in rice cultivation [10]. When these animals fall ill, they lose their utility for farm work, prompting owners to sell them rapidly to mitigate financial loss [31]. Financial survival often takes precedence over disease prevention, particularly since 81.3% of traders rely on livestock as their primary source of income [32,33].

High-risk consumption habits also persist; 42.4% of respondents reported consuming meat from sick animals, and 14.8% consumed unboiled milk. While proper meat cooking (95.1%) and mosquito net usage (74.1%) were common, these are often general habits rather than specific RVF-preventive measures [26,27].

### 4.4. Structural Barriers and Factors Associated with KAP

Multivariable analysis identified education as the strongest and most consistent predictor of good KAP levels. Higher educational attainment greatly increased the odds of having accurate knowledge and positive attitudes [34]. Geographic distance also serves as a critical structural barrier: 57.9% of traders lived more than 5 km from the nearest veterinary service. While distance may directly influence knowledge and attitudes, it has no significant impact on actual practices, which are driven primarily by economic constraints [19,29].

Financial barriers remain the primary obstacle to seeking professional care, with cost cited by 9.1% of respondents as the reason for not consulting a veterinarian. This mirrors challenges in Sudan and Malawi, where the absence of compensation for livestock losses deters reporting [19,26].

### 4.5. Implications for One Health and Control

The virtual absence of participation in awareness campaigns (5.4%) indicates a significant gap in community engagement. Future interventions must adopt a “One Health” approach, acknowledging the interconnected risks between human, animal, and environmental health [3]. Coordination between public health, veterinary services, and environmental agencies is essential for effective detection and outbreak response [12]. Empowering APPSA personnel and utilising trusted sources like private veterinarians will be crucial for bridging the gap between awareness and safe practice [13,35].

### 4.6. Limitations of the Study

This study has several limitations, including its cross-sectional design which prevents causal inference. Additionally, reliance on self-reported practices may introduce social desirability or recall bias.

## 5. Conclusions

This study highlights critical gaps in RVF knowledge, attitudes, and practices among livestock traders in Madagascar. Overall awareness was strikingly low, and high-risk behaviours persist, driven primarily by economic necessity. Education was the most consistent predictor of adequate knowledge, attitudes and practices. Implementing a multisectoral “One Health” approach is imperative to integrate human, animal, and environmental health. Targeted educational interventions and improved access to affordable veterinary services are essential to bridge the gap between awareness and safe practices, helping to safeguard regional economic resilience.

## Figures and Tables

**Table 1:** Socio-demographic and livestock business characteristics of livestock traders in the Alaotra Mangoro region, Madagascar (N = 406).

Variable	Ambatondrazaka (n = 86)	Amparafaravola (n = 66)	Andilamena (n = 91)	Anosibe An’Ala (n = 70)	Moramanga (n = 93)	Total (N = 406)
**Age group**						
18–29 years	31 (36.0%)	19 (28.8%)	31 (34.1%)	29 (41.4%)	27 (29.0%)	137 (33.7%)
30–39 years	23 (26.7%)	16 (24.2%)	30 (33.0%)	20 (28.6%)	24 (25.8%)	113 (27.8%)
40–49 years	20 (23.3%)	15 (22.7%)	17 (18.7%)	11 (15.7%)	31 (33.3%)	94 (23.2%)
50–59 years	12 (14.0%)	12 (18.2%)	9 (9.9%)	9 (12.9%)	10 (10.8%)	52 (12.8%)
≥60 years	0 (0.0%)	4 (6.1%)	4 (4.4%)	1 (1.4%)	1 (1.1%)	10 (2.5%)
**Gender**						
Male	81 (94.2%)	59 (89.4%)	87 (95.6%)	65 (92.9%)	86 (92.5%)	378 (93.1%)
Female	5 (5.8%)	7 (10.6%)	4 (4.4%)	5 (7.1%)	7 (7.5%)	28 (6.9%)
**Education level**						
None	10 (11.6%)	16 (24.2%)	24 (26.4%)	9 (12.9%)	15 (16.1%)	74 (18.2%)
Primary	50 (58.1%)	22 (33.3%)	43 (47.3%)	38 (54.3%)	38 (40.9%)	191 (47.0%)
Secondary	21 (24.4%)	20 (30.3%)	22 (24.2%)	19 (27.1%)	31 (33.3%)	113 (27.8%)
University or college	5 (5.8%)	8 (12.1%)	2 (2.2%)	4 (5.7%)	9 (9.7%)	28 (6.9%)
**Main occupation**						
Trade	52 (60.5%)	45 (68.2%)	56 (61.5%)	38 (54.3%)	73 (78.5%)	264 (65.0%)
Breeding/livestock producer	15 (17.4%)	9 (13.6%)	8 (8.8%)	23 (32.9%)	9 (9.7%)	64 (15.8%)
Butcher	10 (11.6%)	3 (4.5%)	12 (13.2%)	6 (8.6%)	6 (6.5%)	37 (9.1%)
Employee	5 (5.8%)	4 (6.1%)	5 (5.5%)	3 (4.3%)	4 (4.3%)	21 (5.2%)
Crops/agriculture	4 (4.7%)	5 (7.6%)	10 (11.0%)	0 (0.0%)	1 (1.1%)	20 (4.9%)
**Years in business**						
1–9 years	35 (40.7%)	38 (57.6%)	44 (48.4%)	32 (45.7%)	31 (33.3%)	180 (44.3%)
10–19 years	22 (25.6%)	9 (13.6%)	26 (28.6%)	15 (21.4%)	21 (22.6%)	93 (22.9%)
20–29 years	20 (23.3%)	12 (18.2%)	15 (16.5%)	14 (20.0%)	31 (33.3%)	92 (22.7%)
≥30 years	9 (10.5%)	7 (10.6%)	6 (6.6%)	9 (12.9%)	10 (10.8%)	41 (10.1%)
**Trading frequency**						
Occasionally	23 (26.7%)	7 (10.6%)	21 (23.1%)	24 (34.3%)	12 (12.9%)	87 (21.4%)
Once a year	14 (16.3%)	0 (0.0%)	18 (19.8%)	23 (32.9%)	41 (44.1%)	96 (23.6%)
1–15 times per year	26 (30.2%)	48 (72.7%)	31 (34.1%)	8 (11.4%)	27 (29.0%)	140 (34.5%)
16–30 times per year	23 (26.7%)	11 (16.7%)	21 (23.1%)	15 (21.4%)	13 (14.0%)	83 (20.4%)

**Table 2:** Knowledge of RVF among livestock traders by district, Alaotra Mangoro, Madagascar (N = 406).

Variable	Ambatondrazaka (n = 86)	Amparafaravola (n = 66)	Andilamena (n = 91)	Anosibe An’ala (n = 70)	Moramanga (n = 93)	Total (N = 406)
**Heard of RVF**						
Yes	2 (2.3%)	19 (28.8%)	6 (6.6%)	0 (0.0%)	48 (51.6%)	75 (18.5%)
**Source of RVF Information**						
Radio	1 (1.2%)	12 (18.2%)	4 (4.4%)	0 (0%)	22 (23.7%)	39 (9.6%)
Veterinarian	1 (1.2%)	16 (24.2%)	3 (3.3%)	0 (0%)	43 (46.2%)	63 (15.5%)
Family	1 (1.2%)	8 (12.1%)	5 (5.5%)	0 (0%)	38 (40.9%)	52 (12.8%)
Neighbours	1 (1.2%)	7 (10.6%)	5 (5.5%)	0 (0%)	26 (28%)	39 (9.6%)
NGO	0 (0%)	1 (1.5%)	0 (0%)	0 (0%)	0 (0%)	1 (0.2%)
**Mentioned signs of RVF in animals**						
Sudden abortions	1 (1.2%)	16 (24.2%)	6 (6.6%)	0 (0%)	38 (40.9%)	61 (15%)
High fever	0 (0%)	4 (6.1%)	3 (3.3%)	0 (0%)	19 (20.4%)	26 (6.4%)
Weakness	0 (0%)	8 (12.1%)	1 (1.1%)	0 (0%)	12 (12.9%)	21 (5.2%)
Diarrhoea	0 (0%)	6 (9.1%)	2 (2.2%)	0 (0%)	13 (14%)	21 (5.2%)
Nasal discharge	0 (0%)	4 (6.1%)	4 (4.4%)	0 (0%)	30 (32.3%)	38 (9.4%)
Deaths	1 (1.2%)	9 (13.6%)	5 (5.5%)	0 (0%)	40 (43%)	55 (13.5%)
Don’t know	0 (0%)	2 (3%)	0 (0%)	0 (0%)	0 (0%)	2 (0.5%)
**Mentioned signs of RVF in humans**						
High fever	0 (0%)	7 (10.6%)	1 (1.1%)	0 (0%)	11 (11.8%)	19 (4.7%)
Headaches	0 (0%)	4 (6.1%)	2 (2.2%)	0 (0%)	2 (2.2%)	8 (2%)
Muscle pain	0 (0%)	8 (12.1%)	2 (2.2%)	0 (0%)	4 (4.3%)	14 (3.4%)
Blurred vision	0 (0%)	0 (0%)	0 (0%)	0 (0%)	4 (4.3%)	4 (1%)
Haemorrhage	1 (1.2%)	4 (6.1%)	4 (4.4%)	0 (0%)	29 (31.2%)	38 (9.4%)
Back pain	0 (0%)	3 (4.5%)	1 (1.1%)	0 (0%)	1 (1.1%)	5 (1.2%)
Deaths	0 (0%)	2 (3%)	3 (3.3%)	0 (0%)	24 (25.8%)	29 (7.1%)
Don’t know	1 (1.2%)	8 (12.1%)	2 (2.2%)	0 (0%)	5 (5.4%)	16 (3.9%)
**Mentioned modes of RVF transmission to humans**						
Mosquito bite	0 (0%)	1 (1.5%)	1 (1.1%)	0 (0%)	3 (3.2%)	5 (1.2%)
Contact with blood or fluids of infected animals	0 (0%)	9 (13.6%)	1 (1.1%)	0 (0%)	16 (17.2%)	26 (6.4%)
Contact with an aborted foetus	0 (0%)	10 (15.2%)	1 (1.1%)	0 (0%)	25 (26.9%)	36 (8.9%)
Drinking raw milk	1 (1.2%)	8 (12.1%)	4 (4.4%)	0 (0%)	21 (22.6%)	34 (8.4%)
Eating undercooked (raw) meat	2 (2.3%)	10 (15.2%)	4 (4.4%)	0 (0%)	31 (33.3%)	47 (11.6%)
Don’t know	0 (0%)	6 (9.1%)	2 (2.2%)	0 (0%)	7 (7.5%)	15 (3.7%)
**Mentioned seasonality of RVF occurrence**						
I don’t know	2 (2.3%)	10 (15.2%)	3 (3.3%)	0 (0%)	23 (24.7%)	38 (9.4%)
Rainy season	0 (0%)	8 (12.1%)	3 (3.3%)	0 (0%)	25 (26.9%)	36 (8.9%)
Dry season	0 (0%)	1 (1.5%)	0 (0%)	0 (0%)	0 (0%)	1 (0.2%)
**Adaptation of practices during high-risk periods**						
No	0 (0%)	18 (27.3%)	3 (3.3%)	0 (0%)	10 (10.8%)	31 (7.6%)
Yes	0 (0%)	1 (1.5%)	0 (0%)	0 (0%)	15 (16.1%)	16 (3.9%)

**Table 3:** Attitudes towards RVF among livestock traders by district, Alaotra Mangoro, Madagascar (N = 406).

Variable	Ambatondrazaka (n = 86)	Amparafaravola (n = 66)	Andilamena (n = 91)	Anosibe An’ala (n = 70)	Moramanga (n = 93)	Total (N = 406)	*p*-Value *
**Belief in RVF as a real disease**							0.007
No	43 (50%)	34 (51.5%)	42 (46.2%)	41 (58.6%)	29 (31.2%)	189 (46.6%)	
Yes	43 (50%)	32 (48.5%)	49 (53.8%)	29 (41.4%)	64 (68.8%)	217 (53.4%)	
**Perceived danger of RVF**							<0.001
None	19 (22.1%)	17 (25.8%)	20 (22%)	11 (15.7%)	9 (9.7%)	76 (18.7%)	
Little	24 (27.9%)	16 (24.2%)	21 (23.1%)	32 (45.7%)	11 (11.8%)	104 (25.6%)	
Moderate	26 (30.2%)	16 (24.2%)	30 (33%)	25 (35.7%)	29 (31.2%)	126 (31%)	
Very dangerous	17 (19.8%)	17 (25.8%)	20 (22%)	2 (2.9%)	44 (47.3%)	100 (24.6%)	
**Belief in animal-to-human transmission**							<0.001
Don’t know	31 (36%)	14 (21.2%)	32 (35.2%)	30 (42.9%)	15 (16.1%)	122 (30%)	
No	20 (23.3%)	22 (33.3%)	13 (14.3%)	15 (21.4%)	18 (19.4%)	88 (21.7%)	
Yes	35 (40.7%)	30 (45.5%)	46 (50.5%)	25 (35.7%)	60 (64.5%)	196 (48.3%)	
**Perception of personal risk**							<0.001
Don’t know	28 (32.6%)	10 (15.2%)	37 (40.7%)	33 (47.1%)	21 (22.6%)	129 (31.8%)	
No	34 (39.5%)	40 (60.6%)	19 (20.9%)	20 (28.6%)	24 (25.8%)	137 (33.7%)	
Yes	24 (27.9%)	16 (24.2%)	35 (38.5%)	17 (24.3%)	48 (51.6%)	140 (34.5%)	
**Vaccination of livestock against diseases**							<0.001
No	5 (5.8%)	5 (7.6%)	1 (1.1%)	29 (41.4%)	5 (5.4%)	45 (11.1%)	
Yes	81 (94.2%)	61 (92.4%)	90 (98.9%)	41 (58.6%)	88 (94.6%)	361 (88.9%)	
**Confidence in animal vaccination**							<0.001
Uncertain	23 (26.7%)	17 (25.8%)	16 (17.6%)	41 (58.6%)	21 (22.6%)	118 (29.1%)	
No	9 (10.5%)	14 (21.2%)	5 (5.5%)	12 (17.1%)	3 (3.2%)	43 (10.6%)	
Yes	54 (62.8%)	35 (53%)	70 (76.9%)	17 (24.3%)	69 (74.2%)	245 (60.3%)	
**Belief that livestock trade contributes to RVF spread**							<0.001
Uncertain	39 (45.3%)	25 (37.9%)	42 (46.2%)	45 (64.3%)	22 (23.7%)	173 (42.6%)	
No	27 (31.4%)	24 (36.4%)	22 (24.2%)	21 (30%)	39 (41.9%)	133 (32.8%)	
Yes	20 (23.3%)	17 (25.8%)	27 (29.7%)	4 (5.7%)	32 (34.4%)	100 (24.6%)	

* *p*-value for difference between districts.

**Table 4:** Preventive practices and high-risk behaviours related to Rift Valley fever among livestock traders by district (N = 406).

Variable	Ambatondrazaka (n = 86)	Amparafaravola (n = 66)	Andilamena (n = 91)	Anosibe An’ala (n = 70)	Moramanga (n = 93)	Total (N = 406)	*p*-Value *
**Protective measures when handling sick animals**							<0.001
No	83 (96.5%)	62 (93.9%)	90 (98.9%)	70 (100%)	79 (84.9%)	384 (94.6%)	
Yes	3 (3.5%)	4 (6.1%)	1 (1.1%)	0 (0%)	14 (15.1%)	22 (5.4%)	
***(If Yes)*** **Types of protection used**							0.084
Gloves	3 (3.5%)	4 (6.1%)	1 (1.1%)	0 (0%)	14 (15.1%)	22 (5.4%)	
Boots	1 (1.2%)	0 (0%)	0 (0%)	0 (0%)	0 (0%)	1 (0.2%)	
**Mosquito net usage**							0.004
No	21 (24.4%)	15 (22.7%)	36 (39.6%)	9 (12.9%)	24 (25.8%)	105 (25.9%)	
Yes	65 (75.6%)	51 (77.3%)	55 (60.4%)	61 (87.1%)	69 (74.2%)	301 (74.1%)	
**(** ***If no*** **) Reasons for not using nets**							<0.001
Heat/discomfort	17 (19.8%)	0 (0%)	21 (23.1%)	7 (10%)	14 (15.1%)	59 (14.5%)	
Habit/personal preference	16 (18.6%)	0 (0%)	18 (19.8%)	9 (12.9%)	11 (11.8%)	54 (13.3%)	
Low perceived presence of mosquitoes	2 (2.3%)	0 (0%)	2 (2.2%)	0 (0%)	5 (5.4%)	9 (2.2%)	
Use of other means of protection	4 (4.7%)	0 (0%)	14 (15.4%)	7 (10%)	8 (8.6%)	33 (8.1%)	
Lack of stratified mosquito net	0 (0%)	0 (0%)	2 (2.2%)	1 (1.4%)	1 (1.1%)	4 (1%)	
**Avoidance of touching aborted foetuses**							<0.001
No	80 (93%)	55 (83.3%)	84 (92.3%)	67 (95.7%)	69 (74.2%)	355 (87.4%)	
Yes	6 (7%)	11 (16.7%)	7 (7.7%)	3 (4.3%)	24 (25.8%)	51 (12.6%)	
**(** ***If Yes*** **) Methods used to avoid contact**							0.06
Use of protective equipment (e.g., gloves)	2 (2.3%)	3 (4.5%)	1 (1.1%)	0 (0%)	3 (3.2%)	9 (2.2%)	
Contactless burial of foetus	1 (1.2%)	2 (3.0%)	1 (1.1%)	0 (0%)	11 (11.8%)	15 (3.7%)	
Delegation to others	3 (3.5%)	1 (1.5%)	2 (2.2%)	3 (4.3%)	9 (9.7%)	18 (4.4%)	
To avoid disease or illness	0 (0%)	3 (4.5%)	3 (3.3%)	0 (0%)	1 (1.1%)	7 (1.7%)	
Other/unspecified	0 (0%)	1 (1.5%)	0 (0%)	0 (0%)	0 (0%)	1 (0.2%)	
**Distance of animal shelters from house**							<0.001
Animals sleep in the house	3 (3.5%)	4 (6.1%)	4 (4.4%)	7 (10%)	1 (1.1%)	19 (4.7%)	
Less than 5 m	9 (10.5%)	7 (10.6%)	10 (11%)	3 (4.3%)	5 (5.4%)	34 (8.4%)	
Between 5 and 10 m	24 (27.9%)	14 (21.2%)	19 (20.9%)	38 (54.3%)	19 (20.4%)	114 (28.1%)	
More than 10 m	49 (57%)	41 (62.1%)	57 (62.6%)	22 (31.4%)	68 (73.1%)	237 (58.4%)	
I don’t know	1 (1.2%)	0 (0%)	1 (1.1%)	0 (0%)	0 (0%)	2 (0.5%)	
**Consumption of unboiled (raw) milk**							<0.001
No	71 (82.6%)	57 (86.4%)	86 (94.5%)	46 (65.7%)	86 (92.5%)	346 (85.2%)	
Yes	15 (17.4%)	9 (13.6%)	5 (5.5%)	24 (34.3%)	7 (7.5%)	60 (14.8%)	
**Avoidance of animal blood or fluids**							0.004
No	81 (94.2%)	53 (80.3%)	86 (94.5%)	65 (92.9%)	89 (95.7%)	374 (92.1%)	
Yes	5 (5.8%)	13 (19.7%)	5 (5.5%)	5 (7.1%)	4 (4.3%)	32 (7.9%)	
**Willingness to report sick or dead animals to the authorities**							<0.001
No	15 (17.4%)	11 (16.7%)	10 (11%)	32 (45.7%)	11 (11.8%)	79 (19.5%)	
Yes	48 (55.8%)	29 (43.9%)	58 (63.7%)	7 (10%)	54 (58.1%)	196 (48.3%)	
**Slaughtering of sick animals**							0.078
No	51 (59.3%)	34 (51.5%)	58 (63.7%)	31 (44.3%)	58 (62.4%)	232 (57.1%)	
Yes	35 (40.7%)	32 (48.5%)	33 (36.3%)	39 (55.7%)	35 (37.6%)	174 (42.9%)	
**(** ***If yes*** **) Purpose when slaughtering sick animals**							<0.001
For sale/consumption	34 (39.5%)	32 (48.5%)	33 (36.3%)	39 (55.7%)	35 (37.6%)	173 (42.6%)	
Eat yourself	11 (12.8%)	6 (9.1%)	13 (14.3%)	26 (37.1%)	7 (7.5%)	63 (15.5%)	
Burial	1 (1.2%)	0 (0%)	0 (0%)	0 (0%)	0 (0%)	1 (0.2%)	
**Consumption of meat from sick animals**							0.004
No	51 (59.3%)	32 (48.5%)	64 (70.3%)	30 (42.9%)	57 (61.3%)	234 (57.6%)	
Yes	35 (40.7%)	34 (51.5%)	27 (29.7%)	40 (57.1%)	36 (38.7%)	172 (42.4%)	
**Cooking meat properly to avoid illness**							0.001
No	2 (2.3%)	4 (6.1%)	1 (1.1%)	10 (14.3%)	3 (3.2%)	20 (4.9%)	
Yes	84 (97.7%)	62 (93.9%)	90 (98.9%)	60 (85.7%)	90 (96.8%)	386 (95.1%)	
**Selling or buying sick cattle**							<0.001
No	44 (51.2%)	22 (33.3%)	52 (57.1%)	27 (38.6%)	60 (64.5%)	205 (50.5%)	
Yes	42 (48.8%)	44 (66.7%)	39 (42.9%)	43 (61.4%)	33 (35.5%)	201 (49.5%)	

* *p*-value for difference between districts.

**Table 5:** Perception of the preventive role of government, NGOs, and trust in veterinary information sources among livestock traders (N = 406).

Variable	Ambatondrazaka (n = 86)	Amparafaravola (n = 66)	Andilamena (n = 91)	Anosibe An’ala (n = 70)	Moramanga (n = 93)	Total (N = 406)	*p*-Value *
**Do you trust RVF information reported by**							<0.001
Government	43 (50%)	23 (34.8%)	40 (44%)	19 (27.1%)	30 (32.3%)	155 (38.2%)	
Private veterinary Services	73 (84.9%)	42 (63.6%)	71 (78%)	36 (51.4%)	90 (96.8%)	312 (76.8%)	
NGOs	13 (15.1%)	11 (16.7%)	14 (15.4%)	5 (7.1%)	11 (11.8%)	54 (13.3%)	
Not sure	8 (9.3%)	10 (15.2%)	13 (14.3%)	25 (35.7%)	3 (3.2%)	59 (14.5%)	
None	4 (4.7%)	13 (19.7%)	4 (4.4%)	8 (11.4%)	0 (0%)	29 (7.1%)	
**Participation in RVF training or awareness campaign**							<0.001
No	86 (100%)	59 (89.4%)	91 (100%)	69 (98.6%)	79 (84.9%)	384 (94.6%)	
Yes	0 (0%)	7 (10.6%)	0 (0%)	1 (1.4%)	14 (15.1%)	22 (5.4%)	
**(** ***If Yes*** **) Organisers of RVF training or awareness campaign**							<0.001
Government	0 (0%)	0 (0%)	0 (0%)	1 (1.4%)	0 (0%)	1 (0.2%)	
Veterinary services	0 (0%)	7 (10.6%)	0 (0%)	0 (0%)	14 (15.1%)	21 (5.2%)	

* *p*-value for difference between districts.

**Table 6:** Access to professional veterinary services and Proximity Agents (APPSA) among livestock traders by district (N = 406).

Variable	Ambatondrazaka (n = 86)	Amparafaravola (n = 66)	Andilamena (n = 91)	Anosibe An’ala (n = 70)	Moramanga (n = 93)	Total (N = 406)	*p*-Value *
**Distance to nearest veterinary/APPSA service**							<0.001
<1 km	6 (7%)	19 (28.8%)	14 (15.4%)	1 (1.4%)	0 (0%)	40 (9.9%)	
1–5 km	29 (33.7%)	25 (37.9%)	38 (41.8%)	10 (14.3%)	29 (31.2%)	131 (32.3%)	
>5 km	51 (59.3%)	22 (33.3%)	39 (42.9%)	59 (84.3%)	64 (68.8%)	235 (57.9%)	
**Frequency of bringing animals for consultation in case of illness**							<0.001
Never	13 (15.1%)	17 (25.8%)	11 (12.1%)	29 (41.4%)	8 (8.6%)	78 (19.2%)	
Sometimes	49 (57%)	25 (37.9%)	49 (53.8%)	41 (58.6%)	53 (57%)	217 (53.4%)	
Always	24 (27.9%)	24 (36.4%)	31 (34.1%)	0 (0%)	32 (34.4%)	111 (27.3%)	
**(If never) Reasons for not consulting a veterinarian in case of animal illness**							
Too expensive/Lack of financial means	8 (9.3%)	11 (16.7%)	3 (3.3%)	13 (18.6%)	2 (2.2%)	37 (9.1%)	
Too far	0 (0.0%)	2 (3.0%)	1 (1.1%)	5 (7.1%)	2 (2.2%)	10 (2.5%)	
Lack of trust/confidence in vets	0 (0.0%)	3 (4.5%)	2 (2.2%)	3 (4.3%)	0 (0.0%)	8 (2.0%)	
Self-treatment or animal heals on its own	3 (3.5%)	0 (0.0%)	1 (1.1%)	0 (0.0%)	3 (3.3%)	7 (1.7%)	
Sick animals are sold/slaughtered	3 (3.5%)	0 (0.0%)	2 (2.2%)	0 (0.0%)	0 (0.0%)	5 (1.2%)	
Other reasons	2 (2.3%)	0 (0.0%)	2 (2.2%)	3 (4.3%)	0 (0.0%)	7 (1.7%)	
**Place of consultation (among those who always consulted)**							
APPSA and veterinarian (both)	5 (5.8%)	0 (0%)	1 (1.1%)	0 (0%)	2 (2.2%)	8 (2%)	
Veterinarian only	19 (22.1%)	24 (36.4%)	30 (33%)	0 (0%)	30 (32.3%)	103 (25.4%)	

* *p*-value for difference between districts.

**Table 7:** Distribution of reported economic losses and market uncertainty attributed to Rift Valley fever by district (N = 406).

Variable	Ambatondrazaka (n = 86)	Amparafaravola (n = 66)	Andilamena (n = 91)	Anosibe An’ala (n = 70)	Moramanga (n = 93)	Total (N = 406)	*p*-Value *
**Experienced economic losses**							0.001
No	68 (79.1%)	51 (77.3%)	88 (96.7%)	53 (75.7%)	72 (77.4%)	332 (81.8%)	
Yes	18 (20.9%)	15 (22.7%)	3 (3.3%)	17 (24.3%)	21 (22.6%)	74 (18.2%)	
**(** ***If yes*** **) Type of loss (among affected)**							0.075
Animal deaths	16 (18.6%)	14 (21.2%)	3 (3.3%)	17 (24.3%)	15 (16.1%)	65 (16%)	
Decline in income	10 (11.6%)	3 (4.5%)	1 (1.1%)	1 (1.4%)	6 (6.5%)	21 (5.2%)	
Market bans	9 (10.5%)	5 (7.6%)	1 (1.1%)	0 (0%)	7 (7.5%)	22 (5.4%)	
Theft	0 (0%)	0 (0%)	0 (0%)	3 (4.3%)	0 (0%)	3 (0.7%)	

* *p*-value for difference between districts.

**Table 8:** Multivariable analysis of factors associated with Knowledge, Attitudes and Practices regarding Rift Valley fever.

	KNOWLEDGE			ATTITUDE			PRACTICE		
Factors	aOR	95% CI	*p*-Value	aOR	95% CI	*p*-Value	aOR	95% CI	*p*-Value
Gender (male vs. female)	1.2	0.3–4.9	0.780	1.7	0.5–6.1	0.400	3.09	0.34–27.81	0.310
Education									
None	1 *			1 *			1 *		
Primary	1.1	0.4–3.2	0.900	3.3	1.1–9.9	0.039	1.7	0.2–14.9	0.650
Secondary	8.4	2.8–24.8	<0.001	10.4	3.4–32.3	<0.001	8.2	1.0–64.1	0.045
Higher	181.6	29.9–1123.7	<0.001	233.7	45.6–1196	<0.001	45.8	5.2–404.8	<0.001
Household size (number of members)	1.1	0.93–1.4	0.200	1.2	1.0–1.4	0.055	0.9	0.7–1.2	0.530
Age									
18–29 years	1 *			1 *			1 *		
30–39 years	1.2	0.5–3.4	0.671	0.5	0.2–1.1	0.100	0.4	0.1–1.7	0.218
40–49 years	1.79	0.6–5.4	0.302	0.9	0.4–2.1	0.797	0.7	0.2–2.7	0.589
50–59 years	4.4	1.3–15.2	0.019	1.4	0.5–3.8	0.537	2.6	0.6–11.1	0.196
≥60 years	2.4	0.3–18.9	0.396	2.2	0.4–12.4	0.374	12.6	1.5–108.3	0.021
Distance to vet									
<1 km	1 *			1 *			1 *		
1–5 km	0.1	(0.0–0.5)	0.002	0.6	(0.2–1.5)	0.252	0.6	(0.1–2.8)	0.516
>5 km	0.1	(0.0–0.3)	<0.001	0.3	(0.1–0.9)	0.036	0.3	(0.1–1.6)	0.170

* Reference category.

## Data Availability

The datasets used and/or analysed during the current study are available in the UP-Research Data Repository (Figshare), under the Research Data link (Supplementary Materials): https://figshare.com/s/008464250f3fbaa5467c, accessed on 30 January 2026.

## Supplementary Materials

The following supporting information can be downloaded at https://figshare.com/s/008464250f3fbaa5467c. The repository includes the informed consent form, the interview guide used to assess knowledge, attitudes and practices regarding Rift Valley fever among livestock traders, and ethical approval documents.

## Abbreviations

The following abbreviations are used in this manuscript:

ACSA	Community Animal Health Agents (Agents Communautaires de Santé Animale)
APPSA	Proximity Agents in Animal Production and Health (Agents de Proximité en Production et en Santé Animale)
AVSF	Agronomists and Veterinarians Without Borders (Agronomes et vétérinaires sans frontières)
aOR	Adjusted Odds Ratio
CEER	Ethical Evaluation Committee for Biomedical Research (Comité d’Évaluation Éthique pour la Recherche biomédicale)
COI	Indian Ocean Commission (Commission de l’Océan Indien)
DVTD	Department of Veterinary Tropical Diseases
FAO	Food and Agriculture Organisation of the United Nations
GLMMs	Generalised Linear Mixed-Effects Models
GOH	Global One Health
INSPC	National Institute of Public and Community Health (Institut National de Santé Publique et Communautaire)
ITM	Institute of Tropical Medicine
KAP	Knowledge, attitudes and practices
PPE	Personal Protective Equipment
RVF	Rift Valley fever
RVFV	Rift Valley fever virus
VIF	Variance Inflation Factor
